# Impact of ultrasound-assisted extraction on the functional and structural properties, digestibility, hypoglycemic and lipid-lowering effects of seabuckthorn seed meal glutelin

**DOI:** 10.1016/j.fochx.2025.102472

**Published:** 2025-04-16

**Authors:** Wenxia Dong, Jinmei Zhao, Tianyu Zhang, Shilong Xiao, Xinyu Wang, Yang Bi, Juan Wei

**Affiliations:** College of Food Science and Engineering, Gansu Agricultural University, Lanzhou 730070, China

**Keywords:** *Hippophae rhamnoides* L., Non-thermal technology, Protein extraction, Protein conformation, Protein bioactivity

## Abstract

Seabuckthorn seed meal (SSM) is a protein-rich by-product of the oil extraction industry. This study investigated the effects of ultrasound-assisted extraction (UAE) on the physicochemical and functional properties, in vitro digestibility, as well as hypoglycemic and lipid-lowering activities of SSM glutelin. Results indicated that SSM glutelin extracted with UAE (USBG) exhibited higher extraction yield and protein content than SSM glutelin extracted without ultrasound (SBG). Additionally, UAE increased amino acid content, loosened the protein structure, reduced particle size, altered the secondary and tertiary structures of USBG. Moreover, USBG demonstrated enhanced functional properties, like solubility, water-holding and oil-holding capacity, thermal stability, and so on. UAE also improved the in vitro digestibility of USBG, while decreasing the particle size and increasing amino content after digestion. Furthermore, USBG exhibited enhanced hypoglycemic and lipid-lowering activities. Therefore, UAE significantly improves the structure, functionality, digestibility and bioactivities of SSM glutelin, thereby adding value to seabuckthorn by-products.

## Highlights

• Ultrasonic-assisted extraction (UAE) improves glutelin's extraction efficiency.
• UAE reduces particle size, alters secondary and tertiary structures of glutelin.
• UAE improves solubility, emulsion and thermal stability of glutelin.
• UAE enhances glutelin's in vitro digestibility and alters its amino acid composition.
• Hypoglycemic and lipid-lowering activities of glutelin are enhanced by UAE.

## Graphical abstract

**Figure:**
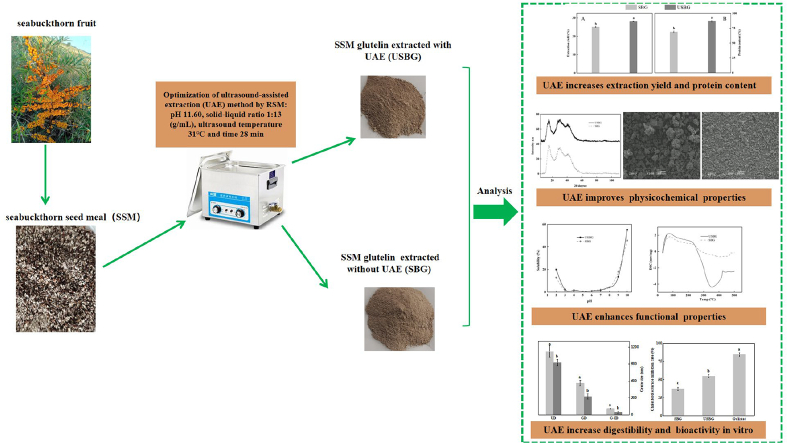

## 1 Introduction

A protein is an organic macromolecule that makes up the fundamental building blocks of cells and functions as a component of food. Glutelin is one of the most important storage proteins in the plant kingdom. Its unique physicochemical properties are characterized by low solubility in neutral solutions, but it easily dissolves in dilute acid and alkali solutions (Ortege et al., 2024). This distinctive solubility gives glutelin its viscoelastic properties, which are of key application value in the food processing industry (Li et al., 2021). Notably, a study by Oluwajuyitan et al. (2024) revealed that glutelin is rich in branched-chain amino acids (including essential amino acids such as leucine, isoleucine, and valine). These amino acids not only participate in protein synthesis but also play an important role in diabetes prevention through physiological mechanisms that promote insulin secretion.

Currently, among the various extraction methods for glutelin, ultrasound-assisted extraction (UAE) is an environmentally friendly green technology (Zhou et al., 2024). Ultrasound-induced cavitation and mechanical effects can alter particle size, protein interactions, structure, and function of protein (Huang et al., 2017). Further, UAE is simple to operate, cost-effective, and could improve mass transfer, reducing solvent consumption and energy consumption, and shortening the extraction time, so it has potential for large-scale industrial and commercial applications. Ortege et al. (2024) reported that UAE could improve both the extraction yield and protein content of olive leaf glutelin. Protein isolated from pumpkin seeds using UAE exhibited a smaller particle size, enhanced solubility, higher surface hydrophobicity, and improved foaming capacity (Du et al., 2022). Furthermore, UAE significantly increased the yield and recovery rate of pepsin-soluble collagen extracted from the skin of Asian bullfrogs while preserving its molecular integrity (Indriani et al., 2023). Additionally, it enhanced the antioxidant activity and bioactivity of collagen hydrolysates produced through papain hydrolysis (Indriani et al., 2022).

Seabuckthorn (*Hippophae rhamnoides* L.) is a deciduous shrub or small tree belonging to the *Elaeagnaceae* family (Wang et al., 2021). In the processing of seabuckthorn seed oil, seabuckthorn seed meal (SSM) is a by-product that contains up to 20 % protein (Xiang et al., 2020). As an excellent dietary protein, seabuckthorn seed protein contains all essential and semi-essential amino acids. Therefore, accelerating in-depth research on SSM protein is crucial for achieving comprehensive utilization of seabuckthorn resources. Enzymatic methods (Zhu et al., 2023), deep eutectic solvent methods, and alkaline methods (Lin et al., 2022) have been used to extract SSM proteins. However, there are currently no reports on the use of UAE for the separation and extraction of SSM glutelin, nor on its effects on the structural and functional properties of SSM glutelin in vitro. Therefore, in this study, the effects of UAE on the structure, functional properties, in vitro digestibility, hypoglycemic and lipid-lowering activities of SSM glutelin were investigated. Building on this theoretical foundation, SSM glutelin can be further explored and applied in depth.

## 2 Materials and methods

### 2.1 Raw materials and reagents

SSM was provided by Gansu Longyuanhong Biotechnology Co., Ltd. After being crushed with a high-speed multipurpose crusher (model 800 Y, voltage 220 V, power 1600 W, frequency 50 Hz, and crushing degree of 30–300 mesh) and passing through a 40-mesh screen, SSM powder was obtained.

The protein quantification assay kit was purchased from Nanjing Jiancheng Bioengineering Co., Ltd. (lot no A045–2). The sodium dodecyl sulfate-polyacrylamide gel electrophoresis was sourced from Bi Yuntian Biotechnology Co., Ltd. (Jiangsu, China), while pepsin was obtained from Beijing Solabio Biotechnology Co., Ltd. (Beijing, China). Trypsin (U/mg solid，EC no 9002-07-7) was acquired from Shanghai McLin Biochemical Technology Co., Ltd. (Shanghai, China), and pancreatic lipase (U/mg solid, EC no 9001-62-1) was purchased from Shanghai Aladdin Biochemical Technology Co., Ltd. (Shanghai, China). Additionally, α-amylase (U/mg solid, EC no 9000-90-2), α-glucosidase (U/mg solid, EC no 9001-42-7), and bile salt-activated cholesterol esterase (U/mg solid, EC no 9026-00-0) were supplied by YuanYe Biotechnology Co., Ltd. (Shanghai, China).

### 2.2 Extraction of SSM glutelin

Glutelin was identified as the most abundant protein in SSM according to the Osboren graded extraction method (Supplementary Fig. S1). UAE of SSM glutelin was carried out by the 25–12 DTD ultrasound extractor (Xinzhi Biotechnology, Zhejiang, China) under 576 W, 40 KHz follow the scheme (Supplementary Fig. S2). In the single-factor experiment, the optimization was carried out within the following ranges: pH (8, 9, 10, 11, 12), solid-to-liquid ratio (1:8, 1:10, 1:12, 1:14, 1:16 g/mL), ultrasound time (5, 10, 20, 40, 60 min), and ultrasound temperature (20, 30, 40, 50, 60 °C). The optimal extraction conditions were determined through response surface analysis to be pH 11.60, a solid-to-liquid ratio of 1:13 (g/mL), ultrasound time of 28 min, and ultrasound temperature of 31 °C (Supplementary Table S1, Supplementary Table S2, Supplementary Fig. S3). SSM glutelin extracted with UAE (USBG) was performed by ultrasound water bath under the optimal extraction conditions. SSM powder extraction with distilled water1:13 (g/mL), and the pH was adjusted with 1 mol/L NaOH. Ultrasound for 28 min under the conditions of 576 W and 31 °C, followed by centrifugation at 5000 rpm for 20 min (4 °C). The supernatant was then adjusted to the isoelectric point with 1 mol/L HCl. Finally, USBG was obtained by centrifugation and freeze-drying (Supplementary Fig. S2). Meanwhile, SSM glutelin extracted without ultrasound under the same conditions served as a control (SBG). The protein samples were kept in a freezer at −20 °C.

### 2.3 Determination of protein content

Based on the method of Khanizadeh et al. (2019), protein content of USBG and SBG was quantified using the Kjeldahl method. Both samples were first digested by heating with concentrated sulfuric acid in the presence of catalysts (copper and potassium) to break down the proteins and release nitrogen. After digestion, the resultant digest was cooled and transferred to a Kjeldahl nitrogen analyzer, where it underwent distillation by adding sodium hydroxide. This process converted the nitrogen in the digest to ammonia gas, which was then absorbed in a boric acid solution. Finally, the boric acid-containing solution was titrated with a standardized hydrochloric acid solution to determine the amount of ammonia, which is directly proportional to the protein content in the sample.

### 2.4 Analysis of physicochemical properties

#### 2.4.1 Determination of amino acid composition

Following the method of Vinayashree et al. (2021), 100 mg of USBG or SBG was hydrolyzed with 2 mL of HCl (6 mol/L) for 22 h. After centrifugation at 5000 rpm for 20 min (4 °C) and vortex mixing for 20 min, the supernatant was collected and filtered through 0.22 μm for LC/MS detection. The LC/MS detection conditions are as follows: mobile phase A was a 20 mmol/L ammonium formate aqueous solution (pH = 3), and mobile phase B was a 20 mmol/L ammonium formate aqueous solution (pH = 3) dissolved in acetonitrile/water (9:1). The injection volume was 1 μL, and the column temperature is maintained at 25 °C.

#### 2.4.2 Observation of microstructure

According to the method of Ortege et al. (2024), the morphological characteristics of USBG and SBG were observed by scanning electron microscopy (SEM) (HITACHI, Japan) at 100 times and 500 times after spraying gold.

#### 2.4.3 Analysis of primary structure

Based on the method of Ortege et al. (2024), the primary structure of SSM protein was identified by sodium dodecyl sulfate-polyacrilamide gel electrophoresis (SDS-PAGE). Ten microliters of the USBG and SBG suspension were loaded after being heated to 100 °C for 3 min. A 12 % separating gel and a 5 % stacking gel were utilized for the electrophoresis process. After electrophoresis, staining was performed used Coomassie Brilliant Blue R250 in a solution containing 45 % methanol and 10 % acetic acid. This was subsequently followed by a destaining procedure using a solution of 5 % methanol and 7.5 % acetic acid.

#### 2.4.4 Analysis of secondary structure

Based on the method of Zhao et al. (2018), a fourier transform infrared spectroscopy (FTIR) spectrometer (Thermo Scientific TM, Nicolet iS50) was employed to test the secondary structure of USBG and SBG. The instrument operated at a resolution of 4 cm^−1^, averaging 32 scans per spectrum, with data collected within the 4000–500 cm^−1^ range. The secondary structure was analyzed by Omnic and PeakFit 4.12 software packages.

#### 2.4.5 Evaluation of crystalline properties

Crystalline properties of glutelin were evaluated following the procedure by Sahni et al. (2020), USBG and SBG were scanned at a speed of 1°min^−1^ used an XRD (Panalytical's X'Pert Pro) to ascertain the X-ray diffraction patterns.

#### 2.4.6 Assessment of particle size distribution and zeta potential

Followed by the method of Alavi et al. (2021), The USBG or SBG suspensions were prepared using 1 % phosphate buffer, and the pH values were adjusted to 2, 3, 4, 5, 6, 7, 8, 9, and 10, respectively. Subsequently, their particle size distribution and zeta potential were measured at 25 °C using a BT-zeta100 potential analyzer (Malvern Instruments Ltd., UK).

#### 2.4.7 Evaluation of surface hydrophobicity

According to the method of Shi et al. (2024), 0.25 g of USBG or SBG was dissolved in 10 mL of phosphate buffer. After centrifugation at 5000 rpm for 10 min (4 °C) and collection of the supernatant, the sample concentrations were diluted to 1, 2, 3, 4, and 6 mg/mL. 50 μL of ANS solution was then added, and the mixture was left to stand in the dark for 15 min. Fluorescence intensity was measured using an F-4700 fluorescence spectrophotometer (Hitachi, Japan) at excitation (400 nm) and emission (690 nm) wavelengths. The slit width was set at 10 nm. A plot of protein mass concentration on the horizontal axis against fluorescence intensity on the vertical axis produced an equation, with the slope indicating surface hydrophobicity.

#### 2.4.8 Determination of free sulfhydryl group and disulfide bond

The free sulfhydryl group and disulfide bond were determined according to the method of Cui et al. (2023). To determine sulfhydryl group, 60 mg of USBG or SBG was dissolved in 5 mL of Tris-Gly-urea buffer. After adding 50 μL of Ellman's reagent, the solution was centrifuged at 3000 rpm for 10 min (4 °C) after 1 h of dark incubation. The absorbance of supernatant was measured at 412 nm. To determine total sulfhydryl groups, 120 mg of USBG or SBG was dissolved in 10 mL of Tris-Gly-urea buffer and treated with 40 μL of β-mercaptoethanol for 2 h. Subsequently, 4 mL of 12 % trichloroacetic acid was added, and the mixture was allowed to react for 1 h. After centrifugation at 5000 rpm for 20 min (4 °C), the residue was washed 3 times with 12 % trichloroacetic acid. The absorbance was measured at 412 nm after adding 80 μL Ellman's reagent and 2 mL Tris-Gly-urea solution. The free sulfhydryl content was calculated using formula (1), the disulfide bond content was calculated using formula (2), and the total sulfhydryl content was calculated by adding the free sulfhydryl groups together.

(1) $$\text{Free sulfhydryl content}\;\left(\text{μmol}/g\right)=\frac{73.53•A_{412}•D}{C}$$

(2) $$\text{Disulfide bond content}\;\left(\text{μmol}/g\right)=\frac{\text{Total thiol}-\text{free thiol}}{2}$$

Molar extinction coefficient: 73.53 for Ellman's reagent. C represented the protein concentration (mg/mL), while D denoted the dilution factor.

### 2.5 Analysis of functional properties

#### 2.5.1 Evaluation of solubility

Following the method of Ortege et al. (2024), 1 % USBG or SBG suspension was prepared using a pH 7.2–7.4 phosphate buffer. The pH was adjusted to 2–10 used 1 mol/L NaOH or HCl. 1 % USBG or SBG prepared with 0.1 mol/L NaOH as a control. The supernatant was centrifuging at 5000 rpm for 20 min (4 °C), the samples were shaken for 1 h at 25 °C. The amount of protein was then measured using a protein quantification kit. The solubility of the protein was calculated according to formula (3).

(3) $$\text{Protein soubility}=\frac{P1}{P2}\times 100$$

P1 was the protein content (%) of the sample, and P2 was the protein content (%) of the control.

#### 2.5.2 Determination of water holding capacity (WHC) and oil holding capacity (OHC)

Based on the method of Stone et al. (2015), 0.2 g of USBG and SBG were each weighed, and 3 mL of either deionized water or soybean oil was added. The mixture was stirred for 2 min, then incubate in a water bath at 20 °C for 20 min. After incubation, the samples were centrifuged at 5000 rpm for 20 min (4 °C), the top layer of water or soybean oil was discarded, the substrate and tubes were weighed. WHC was calculated using formula (4), while OHC was determined using formula (5).

(4) $$\mathrm{WHC}=\left(M2-M1\right)/M$$

(5) $$\mathrm{OHC}=\left(M2-M1\right)/M$$

M stood for glutelin sample mass (g), M1 for centrifuge tube mass and protein sample (g), and M2 for centrifuge tube mass and sediment (g).

#### 2.5.3 Evaluation of foam capacity (FC) and foam stability (FS)

Following the method of Ortege et al. (2024), 1 g of USBG or SBG was weighed and mixed with 10 mL of distilled water. Following a high-speed shearing process lasting 3 min, the total volume was recorded to assess the protein's FC. After standing for 30 min, the total volume was measured again to evaluate FS. Formula (6) was used to calculate FC, while formula (7) was used to calculate FS.

(6) $$\;\mathrm{FC}\left(\%\right)=\left[\left(V1-V\right)/V\right]\times 100$$

(7) $$\mathrm{FS}\left(\%\right)=\left(V2/V\right)\times 100$$

V stood for the total volume prior to homogenization, V1 for the total volume following instantaneous homogenization, and V2 for the total volume following 30 min of homogenization.

#### 2.5.4 Examination of emulsifying activity (EA) and emulsion stability (ES)

According to the method of Biswas et al. (2020), 1 % USBG or SBG suspension was prepared by pH 7.2–7.4 phosphate buffer. Each sample was then mixed with 5 mL of soybean oil, wand homogenized for 2 min. The height of the emulsified layer was then measured. The emulsified samples were allowed to stand for 30 min, after which the height of the emulsified layer was measured again. Formula (8) was used to calculate EA, and formula (9) was used to calculate ES.

(8) $$\mathrm{EA}=\left(H1/H\right)\times 100$$

(9) $$\mathrm{ES}=\left(H2/H1\right)\times 100$$

H1 was the initial emulsified layer height, H was the total height, and H2 was the emulsified layer height after 30 min of settling.

#### 2.5.5 Assessment of thermal characteristics and thermal stability

Based on the method of Peng et al. (2024) with a few minor modifications, the thermal characteristics of SSM glutelin were evaluated using differential scanning calorimetry (DSC), and thermal stability was assessed via thermogravimetric analysis (TGA). For DSC analysis, samples were scanned using a DSC 204F1 calorimeter under a nitrogen gas flow, with temperatures ranging from 0 to 500 °C. For TGA assay, samples were scanned within the same temperature range, at a rate of 10 °C/min used a thermogravimetric analyzer (TA Instruments, TGA 550, USA).

### 2.6 Assessment of in vitro protein digestibility

Following the procedure by Jin et al. (2020), 1 % USBG or SBG suspension was prepared using a pH 7.2–7.4 phosphate buffer. 16 mL simulated gastric juice, 0.2 g pepsin, 10 μL CaCl_2_ (0.3 M) was added to the samples, and the pH value was adjusted to 3.0. Samples were then shaken at 37 °Cand 135 rpm for 2 h, pH was adjusted to 7.0 with 1 mol/L NaOH to terminate gastric digestion. The gastric digestibility was calculated according to formula (10).

When testing the intestinal digestibility, the gastric digestive juice was prepared according to the same method as above. Then 16 mL simulated intestinal fluid, 0.2 g trypsin, 0.16 g pig bile salt, 40 μL CaCl_2_ were added to the gastric digestive juice. The pH value was adjusted to 7.0, and the digestion was carried out for 2 h. Intestinal digestion was terminated by water bath at 75 °Cfor 30 min. Intestinal digestibility was calculated according to formula (10).

(10) $$\mathrm{IVPD}\left(\%\right)=\left(C•V\right)/M$$

V was the volume of the digestive juice, M was the mass of the protein, and C was the protein concentration (mg/mL) of the digestive juice after subtracting the blank.

### 2.7 Determination of hypoglycemic activity in vitro

#### 2.7.1 Evaluation of α-glucosidase inhibition rate

With minor modification to the methodology of Fadimu et al. (2022), a mixture of 100 μL USBG or SBG solution and 100 μL α-glucosidase was incubated at 37 °C for 10 min. After adding 100 μL of 4-nitrophenyl α-D-glucopyranoside (PNPG), the sample was further incubated at 37 °C for 30 min. The reaction was then stopped by adding 100 μL of 1 mol/L sodium carbonate, and the absorbance was measured at 405 nm. Acarbose (2 μg/mL) was used as a positive control. The inhibition rate of α-glucosidase was estimated using formula (11).

(11) $$\text{Inhibition rate of}\;\alpha -\text{glucosidase}\;\left(\%\right)=\left[1-\left(\frac{A1-A2}{A3-A4}\right)\right]\times 100$$

Absorbance was measured for: A1 (sample), A2 (background control, PBS instead of enzyme), A3 (negative control, PBS instead of sample), and A4 (negative control background, PBS instead of sample and enzyme).

#### 2.7.2 Evaluation of α-amylase inhibition rate

Following the method of Fadimu et al. (2022), a mixture of 100 μL of USBG or SBG solution, and 100 μL of α-amylase solution was prepared and incubated for 10 min at 37 °C. The starch solution (100 μL) was then introduced and agitated. After another10 min of incubation at 37 °C, 100 μL of 3,5-dinitrosalicylic acid was added, and the mixture was incubated in boiling water for 3 min. A microplate reader was used to determine the absorbance at 540 nm. 0.1 mg/mL of acarbose was used as a positive control. The inhibition rate of α-amylase was determined with formula (11).

### 2.8 Determination of lipid-lowering activity in vitro

#### 2.8.1 Evaluation of pancreatic cholesterol lipase inhibition rate

Following the procedure by Ajayi et al. (2021), 1 mL of phosphate buffer solution containing taurocholic acid sodium salt (5.16 mmol/L) and NaCl (0.1 mol/L), 50 μL of cholesterol esterase, 50 μL of USBG or SBG, and 50 μL of *p*-nitrophenyl butyrate, was mixed and allowed to react for 30 min at 25 °C. The absorbance was then measured at 405 nm. Orlistat served as the positive control, pancreatic cholesterol lipase inhibition rate was calculated by formula (11).

#### 2.8.2 Evaluation of pancreatic lipase inhibition rate

According to the method of Ajayi et al. (2021), 50 μL of USBG or SBG was mixed with 50 μL of PBS and 50 μL of pancreatic lipase solution. Then, 100 μL of lauric acid 4-nitrophenyl ester solution was added, and the mixture was incubated for 20 min at 37 °C. The absorbance was measured at 405 nm. Orlistat was used as a positive control. The pancreatic lipase inhibition rate was calculated using formula (11).

### 2.9 Data processing

Every experiment was conducted three times. To compute the mean and standard deviation (±SD), Excel 2022 was utilized. For significant analysis (*P* < 0.05), SPSS 26.0 was utilized. Use Origin 2021 for drawing.

## 3 Results and discussion

### 3.1 Effects of UAE on extraction yield and protein content of SSM glutelin

**Fig. 1:**
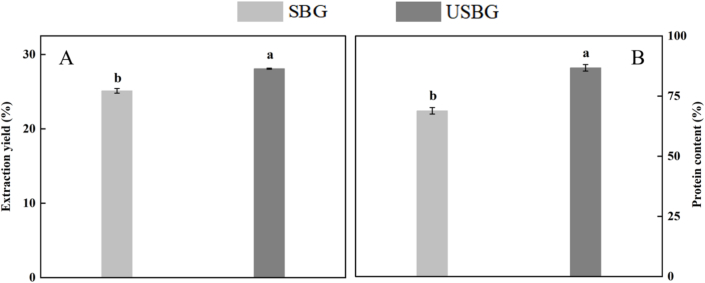
Effects of ultrasound-assisted extraction (UAE) on extraction yield (A) and protein content (B) of SSM glutelin. Different letters indicate significant differences between means (*p < 0.05*), while the standard deviation (±SD) was represented by a vertical line.

The results demonstrated that USBG increased the extraction yield and protein content by 11.87 % and 25.98 % compared to SBG (Fig. 1) (*P < 0.05*). This outcome aligned with the findings of Saha et al. (2016), which reported that UAE can significantly enhance both the protein content and the extraction efficiency of fern leaf protein. Aviation effects generated by ultrasound could help disrupt cell walls and break down protein-lipid or protein-polysaccharide interaction, improving protein release (Rahel et al., 2022). Moreover, ultrasound treatment causes the tight structural regions of glutelin to unfold, exposing the internal hydrophobic groups (Saha et al., 2016), thereby enhancing their interaction with the solvent and ultimately improving solubility, which helps increase the extraction yield. Furthermore, glutelin is typically rich in intramolecular disulfide bonds, and ultrasound may break some of these disulfide bonds, leading to a relaxation of the molecular conformation and further promoting dissolution (Liu et al., 2023). The increase in solubility and the reduction in disulfide bonds observed in the following results support the explanation of the above mechanism.

### 3.2 Effects of UAE on the physicochemical characteristics of SSM glutelin

#### 3.2.1 Influence on amino acid composition

Both USBG and SBG contain 20 amino acids, classifying them as complete proteins. Compared with SBG, USBG exhibited an 8.41 % increase in the total content of hydrophobic amino acids, along with a 6.62 % increasing in total essential amino acids and a 13.14 % increasing in total non-essential amino acids (Table 1) (*P < 0.05*). Li et al. (2017) also found that UAE could increase the total content of hydrophobic amino acids in rice protein by 4.78 %. Additionally, it was noted that the amount of total essential and non-essential amino acids in olive leaf glutelin was raised by 6.98 % and 9.78 %, respectively, by UAE (Ortege et al., 2024). The effect of UAE on the composition of amino acid mainly due to the cavitation effect and mechanical forces generated by ultrasound, which lead to the disintegration of protein aggregates and the unfolding of proteins, thereby exposing previously buried hydrophobic groups and amino acids on the protein surface (Hu et al., 2013; Li et al., 2023).

**Table 1:** Amino acid composition of SSM glutelin.

Amino acid	SBG(mg/100 mg)	USBG(mg/100 mg)
Thr	0.161 ± 0.006^a^	0.167 ± 0.003^a^
Phe	0.156 ± 0.007^a^	0.168 ± 0.167^a^
Lys	0.346 ± 0.036^b^	0.397 ± 0.010^a^
Leu	0.220 ± 0.013^a^	0.235 ± 0.004^a^
Ile	0.117 ± 0.004^a^	0.130 ± 0.003^a^
Trp	ND	ND
Met	0.048 ± 0.005^a^	0.054 ± 0.002^a^
Val	0.139 ± 0.012^a^	0.154 ± 0.154^a^
His	0.172 ± 0.008^a^	0.150 ± 0.020^a^
**EAA**	1.359 ± 0.092^b^	1.455 ± 0.006^a^
Pro	0.181 ± 0.009^a^	0.196 ± 0.002^a^
Tyr	0.189 ± 0.015^a^	0.205 ± 0.004^a^
Cys	0.245 ± 0.004^a^	0.301 ± 0.005^a^
Ala	0.281 ± 0.015^a^	0.301 ± 0.005^a^
Gly	0.529 ± 0.047^a^	0.595 ± 0.012^a^
Ser	1.280 ± 0.107^b^	1.462 ± 0.030^a^
Glu	0.055 ± 0.002^a^	0.082 ± 0.001^a^
Asp	2.428 ± 0.121^b^	2.839 ± 0.047^a^
Arg	0.469 ± 0.012^a^	0.522 ± 0.008^a^
Gln	0.341 ± 0.032^a^	0.397 ± 0,010^a^
Asn	0.103 ± 0.010^a^	0.122 ± 0.002^a^
**NEAA**	6.101 ± 0.361^b^	7.023 ± 0.035^a^

Note: EAA represents total essential amino acids, NEAA represents non-total essential amino acids. ND indicates not detected, and a, b indicate significant differences (*P < 0.05*).

#### 3.2.2 Influence on morphological characteristics and primary structure

**Fig. 2:**
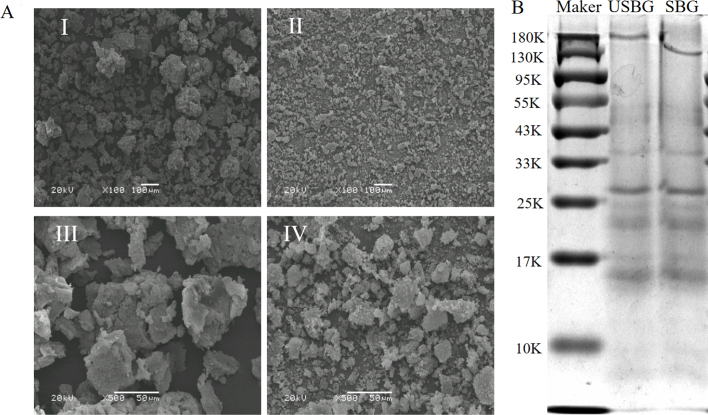
Effects of ultrasound-assisted extraction (UAE) on SSM glutelin in terms of microstructure obtained by Scanning electron microscope (SEM) (A) (I: 100 × magnification for SBG, III: 500 × magnification for SBG, II: 100 × magnification for USBG, IV: 500 × magnification for USBG), and primary structure obtained by Sodium dodecyl sulfate-polyacrilamide gel electrophoresis (SDS-PAGE) profiles (B).

Results of SEM showed that, compared to SBG, USBG has smaller fragments at two different magnifications (100 x, 500 x) and was more evenly dispersed and loosely structured (Fig. 2A). Similar reports also demonstrated that UAE also resulted in more homogeneous and looser dispersion of pumpkin seed glutelin and olive leaf glutelin (Ortege et al., 2024; Du et al., 2022). Ultrasound action breaks down the protein's molecular structure and shrinks the particle size (Du et al., 2022). SDS-PAGE showed that USBG and SBG exhibit some common bands in the ranges of 10–17 KDa, 25–33 KDa, 33–43 KDa, and 130–180 KDa (Fig. 2B), confirming that ultrasound treatment did not significantly alter the compositional ratio of the subunits. Similar outcomes in pea protein following UAE were also observed by Xiong et al. (2018), which were attributed to the fact that ultrasound treatment did not change the protein's fundamental structure.

#### 3.2.3 Influence on secondary structure

**Fig. 3:**
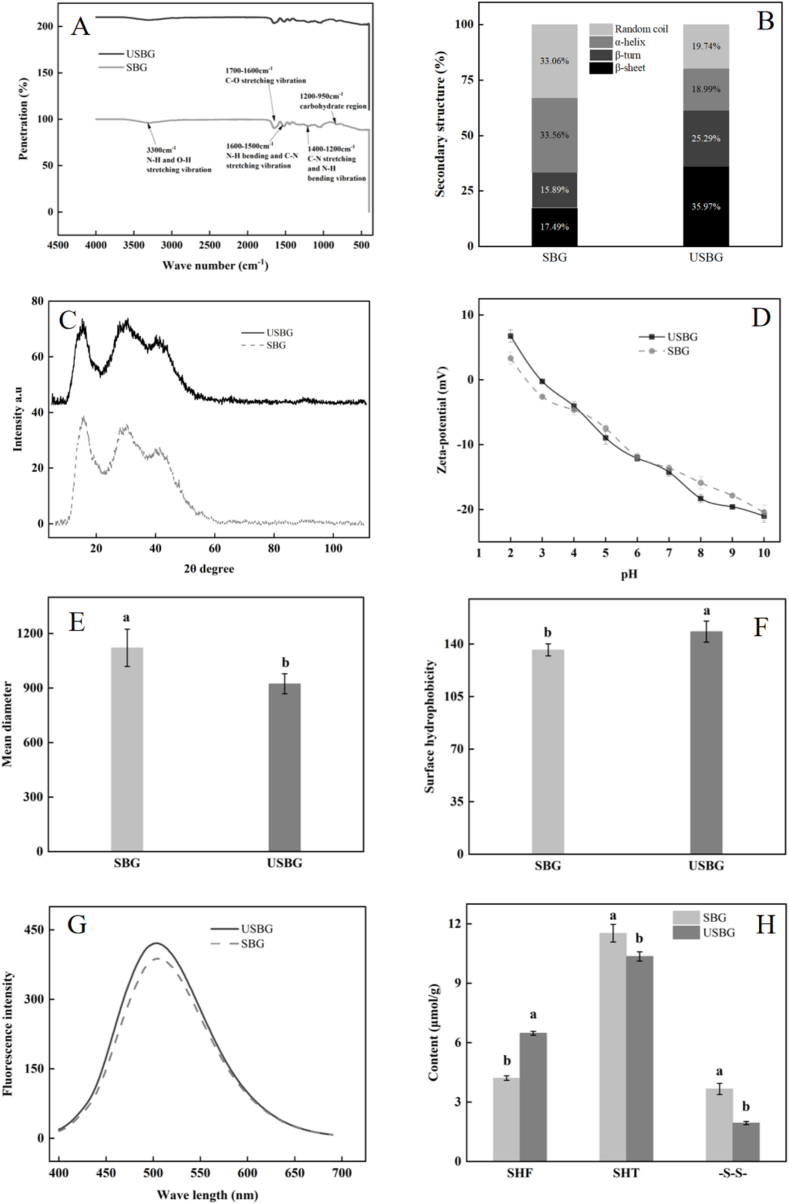
Effects of ultrasound-assisted extraction (UAE) on physicochemical characteristics of SSM glutelin in terms of fourier transform infrared spectroscopy (A), quantification of secondary structure (B), x-ray diffraction (XRD) (C), zeta potential (D), particle size (E), surface hydrophobicity (F), endogenous fluorescence spectrum (G), and content of free thiol groups (SHF), total thiol groups (SHT), disulfide bonds (-S-S-) (H). Different letters indicate significant differences between means (*p < 0.05*), while the standard deviation (±SD) was represented by a vertical line.

The secondary structure of proteins in a physical state can be assessed using the molecular vibrational spectroscopy method known as FTIR (Choi et al., 2005). By looking for adjustments in peak positions close to amide I (1700–1600 cm^−1^), amide II (1600–1500 cm^−1^), and amide III (1400–1200 cm^−1^), the changes in protein structure were examined. In the present study, it was found that USBG and SBG had essentially similar profiles in the 4000–500 cm^−1^ spectral range (Fig. 3A). A correlation between secondary structure and scanning wave number was established using Peakfit to deconvolute the amide I band. The following assignments were made: β-sheet (1610–1640 cm^−1^), random coil (1640–1650 cm^−1^), α-helix (1650–1660 cm^−1^), and β-turn (1660–1700 cm^−1^) (Zhao et al., 2018). Our results showed that USBG increased the β-fold and β-turn content by 51.38 % and 59.16 %, while α-helix and irregular curl decreased by 43.41 % and 40.29 % compared to SBG (Fig. 3B) (*P < 0.05*), which was in line with the trend of UAE that resulted in a decrease in the α-helix and irregular curl content of chickpea proteins (Zhu et al., 2022). Ultrasound can alter the secondary structure of proteins through cavitation, shear forces, and thermal effects. Acoustic cavitation occurs when ultrasound generates microscopic bubbles in a liquid, which collapse violently, creating localized high pressure and temperature, breaking hydrogen bonds and disrupting alpha-helices and beta-sheets. Shear and mechanical forces from rapid ultrasound oscillations cause high shear stress, leading to protein unfolding. Additionally, local heating from ultrasound can destabilize hydrogen bonds, triggering structural transitions in proteins (Rahman et al., 2021).

#### 3.2.4 Influence on crystalline properties

XRD was used to test the crystalline properties of SSM glutelin by determining crystal size and protein conformational changes. The diffraction angle (2θ) and peak intensity provide insights into these characteristics, helping to assess structural modifications in the protein (Sahni et al., 2020). Results showed that the USBG incident angle 2θ had characteristic peaks at 14.70°, 26.44°, 47.94°, and the SBG incident angle 2θ had characteristic peaks at 16.50°, 39.85°, 49.86° (Fig. 3C). UAE caused a decrease in the diffraction angle of SSM glutelin, consistent with its effect on bamboo shoot protein, indicating that UAE can alter the crystalline properties of SSM (Du et al., 2023). This effect is primarily attributed to the cavitation generated by ultrasound, which reduces the crystal size of the protein (Sahni et al., 2020). Furthermore, ultrasound can disrupt intermolecular bonds within proteins, altering the secondary and tertiary structures, which could influence the crystallinity of protein (Fadimu et al., 2022).

#### 3.2.5 Influence on zeta potential and particle size

As pH increased, the zeta potential of both USBG and SBG shifted from positive to negative values. Over the whole pH range, however, USBG continuously maintained a larger zeta potential than SBG (Fig. 3D) (*P < 0.05*). The zeta potential alterations of broad bean protein and sunflower meal protein following UAE were in line with this outcome (Alavi et al., 2021; Dabbour et al., 2021). At the same time, UAE reduced the average particle size of USBG to 923.25 nm, while the value for SBG was 1121.39 nm (Fig. 3E) (*P < 0.05*). A similar study found that UAE reduced the particle size of broad bean protein (Alavi et al., 2021). UAE increases the zeta potential of proteins by exposing buried charged amino acids through protein unfolding, which strengthens the surface charge. It can also induce localized pH changes via water sonolysis, altering the protonation state of amino acids and modifying the zeta potential (Yang et al., 2023). Furthermore, Ultrasound reduces protein particle size by disrupting hydrophobic interactions and weak bonds that hold aggregates together, fragmenting them into smaller, more uniform particles through shear and cavitation effects (Li et al., 2022). Additionally, the increased zeta potential strengthens electrostatic repulsion between protein molecules, preventing aggregation and stabilizing smaller particles (Yang et al., 2023). More importantly, the particle size reduction will enhance some functional properties of protein, such as improved solubility, WHC, OHC (Zhao et al., 2021).

#### 3.2.6 Influence on surface hydrophobicity and endogenous fluorescence spectroscopy

Surface hydrophobicity, which has a major impact on the stability, shape, and function of the protein, is determined by the amount of hydrophobic groups present on the protein surface (Shen et al., 2017). Compared to the surface hydrophobicity of SBG at 136.08, the surface hydrophobicity of USBG increased to 147.91 (Fig. 3F) (*P < 0.05*). Similarly, UAE could increase the surface hydrophobicity of pumpkin seed glutelin and soy protein (Du et al., 2022; Hu et al., 2013). The cavitation and shear forces induced by ultrasound lead to the exposure of hydrophobic amino acid residues buried inside the protein, thereby enhancing its surface hydrophobicity (Liu et al., 2021). By altering the microenvironment of aromatic amino acid residues (tyrosine, phenylalanine, and tryptophan), the tertiary structure of proteins can be detected by endogenous fluorescence spectroscopy (Zhao et al., 2022). Compared to SBG, USBG had a higher fluorescence intensity (Fig. 3G) (*P < 0.05*). According to Jin et al. (2020), buckwheat protein fluorescence intensity may also be increased by UAE. The protein fluorescence intensity was increased by the migration of aromatic amino acid residues, particularly tryptophan residues, to the protein surface as a result of ultrasound treatment (Zhao et al., 2022).

#### 3.2.7 Influence on free sulfhydryl and disulfide bond content

Thiol and disulfide bonds, as secondary linkages, play a crucial role in maintaining the tertiary structure of proteins, facilitating proper folding, and influencing their surface activity. According to our results, USBG's free sulfhydryl concentration rose by 53.68 % when compared to SBG, but its disulfide bond content dropped by 46.99 % (Fig. 3H) (*P < 0.05*). This was like the findings of Liu et al. (2023), which showed that UAE could increase the protein's free sulfhydryl concentration while decreasing its disulfide bond content. According to Jin et al. (2020), the increase in free sulfhydryl content was due to ultrasound-induced exposure of thiol groups within the protein molecules to the surface, as well as the disruption of disulfide bonds.

### 3.3 Effects of UAE on the functional characteristics of SSM glutelin

#### 3.3.1 Influence on solubility

**Fig. 4:**
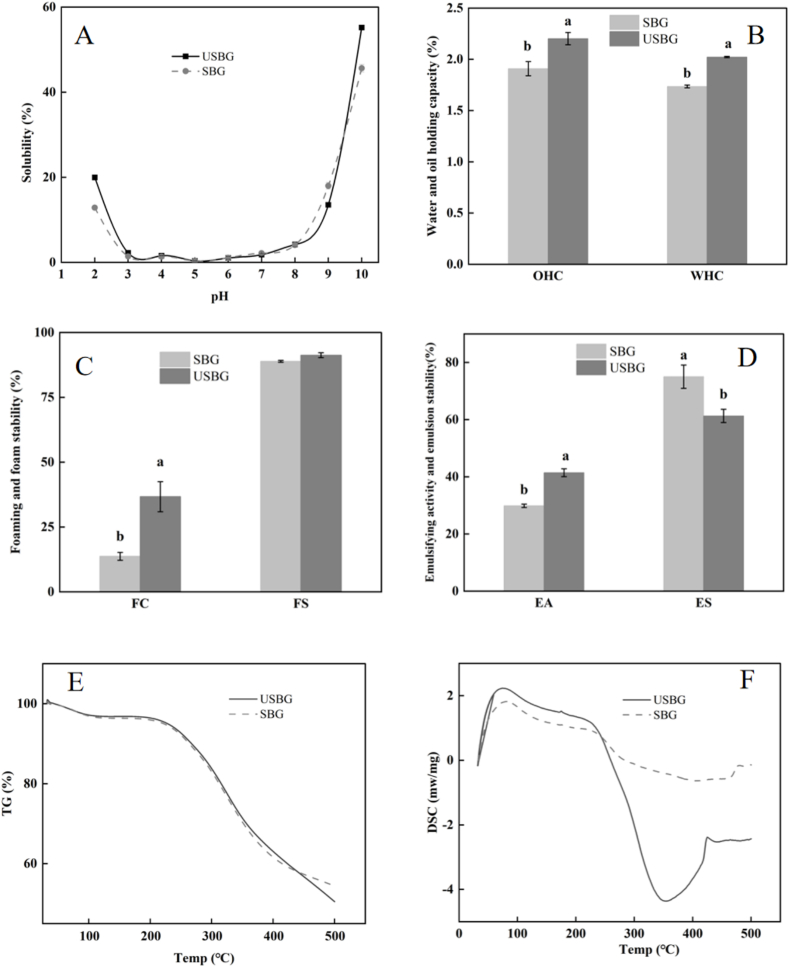
Effects of ultrasound-assisted extraction (UAE) on functional characteristics of SSM glutelin in terms of solubility (A), water holding capacity (WHC) and oil holding capacity (OHC) (B), foaming (FC) and foam stability (FS) (C), emulsifying activity (EA) and emulsion stability (ES) (D), thermogravimetric analysis (E), and differential scanning calorimetry (F). Different letters indicate significant differences between means (*p < 0.05*), while the standard deviation (±SD) was represented by a vertical line.

According to our findings, as the pH level rose, the solubility of USBG and SBG initially dropped and subsequently increased (Fig. 4A). At pH 5, which is close to the isoelectric point of glutelin, the protein exhibited minimal solubility, recorded USBG solubility at 0.36 % and SBG solubility at 0.31 %. Conversely, at pH 10, both USBG and SBG demonstrated peak solubility levels, achieved rates of 55.19 % and 45.62 %, respectively (*P < 0.05*). So, it could be referred that UAE increased the solubility of SSM glutelin which aligned with the solubility increasing effect of UAE on black bean protein and olive leaf glutelin (Ortege et al., 2024; Jiang et al., 2014). The cavitation effect generated during UAE disrupts protein aggregates and facilitates the unfolding of protein structure, exposing hydrophilic groups that enhance water interaction and solubility (Huang et al., 2023). Furthermore, the enhancing effect of UAE on solubility is beneficial to some other functional properties. For example, soluble proteins reduce surface tension and stabilize air bubbles, leading to improved FC (Karaca et al., 2011). Moreover, increased solubility allows proteins to interact better with water, enhancing WHC (Ghribi et al., 2015).

#### 3.3.2 Influence on WHC and OHC

**Fig. 5:**
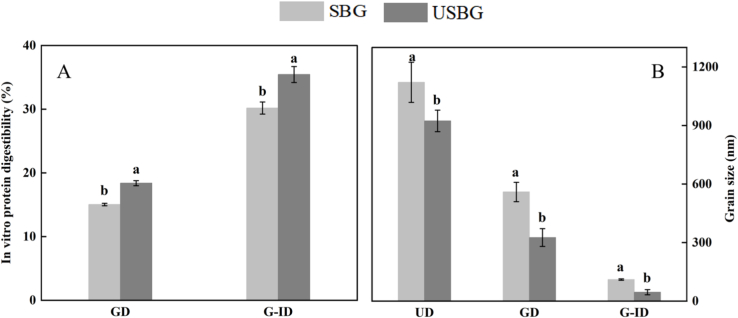
Effects of ultrasound-assisted extraction (UAE) on SSM glutelin in terms of in vitro protein digestibility (A) (GD: gastric digestion, G-ID: gastric digestion followed by intestinal digestion), particle size after in vitro gastric and gastrointestinal digestion (B) (UD represents undigested, GD represents gastric digestion, and G-ID represents both gastric and intestinal digestion). Different letters indicate significant differences between means (*p < 0.05*), while the standard deviation (±SD) was represented by a vertical line.

Our results showed that UAE increased the WHC and OHC of SSM glutelin, with the WHC and OHC of USBG being 29.38 % and 28.66 % higher, respectively, than those of SBG (Fig. 4B) (*P < 0.05*). Other investigations have also shown that UAE can enhance the WHC and OHC of olive leaf and tamarind proteins (Ortege et al., 2024; Biswas et al., 2020). The cavitation effect of ultrasound alters protein structures and causes proteins to unfold, exposing previously hidden hydrophobic and hydrophilic groups. This increased surface area and exposure enhance the protein's ability to interact with both oil and water (Biswas et al., 2020). Hydrophobic groups bind more readily with oils, boosting oil-holding capacity, while hydrophilic groups attract and retain water, increasing water-holding capacity. Additionally, reduced particle size of USBG (Fig. 4E) also enhanced the accessibility of hydrophilic groups, improving WHC, while exposing more hydrophobic regions that interact with oil, boosting OHC (Du et al., 2022). Moreover, increased solubility of USBG (Fig. 5A) improved WHC by increasing protein-water interactions through hydrogen bonding and electrostatic forces (Moure et al., 2006), while enhanced protein dispersion, preventing aggregation and allowing better interaction with oil molecules, leading to higher OHC (Karaca et al., 2011).

#### 3.3.3 Influence on FC and FS

Because of their amphiphilic characteristics (hydrophobic and hydrophilic ends), proteins can stabilize foam (Ortege et al., 2024). According to Fig. 4C, the FC of USBG was 36.67 %, whereas that of SBG was 13.67 % (*P < 0.05*). Similar results about the effects of UAE on foaming qualities of pineapple honey leaf protein and soybean protein were also reported (Jambrak et al., 2009; Julian et al., 2022). The localized high pressure and shear forces generated by the cavitation effect of ultrasound can disrupt the secondary/tertiary structure of proteins. This exposes hydrophobic and hydrophilic group, increasing interfacial activity, then enhances FC (Biswas et al., 2020). Furthermore, reduced particle size and increased solubility of USBG (Fig. 3E, Fig. 4A) facilitate proteins migrate to the air-water interface, stabilize foam, allow for better dispersion of proteins in the continuous phase, which could improve FC (Hu et al., 2013; Jambrak et al., 2009).

#### 3.3.4 Influence on EA and ES

The ability of proteins to facilitate the formation of oil-in-water emulsions is known as EA (Hu et al., 2013). The capacity of proteins to maintain stable emulsions within a given time frame is referred to ES (Arzeni et al., 2012). In this study, USBG demonstrated a 22.4 % drop in ES and a 38.76 % rise in EA when compared to SBG (Fig. 4D) (*P < 0.05*). According to Ortege et al. (2024), the EA of olive leaf glutelin increased by 14.21 % while the ES decreased by 3.56 % when extracted with UAE. UAE induces cavitation effects that can alter protein structures, enhancing their ability to adsorb at the oil–water interface. This increased adsorption improves EA by facilitating the formation of emulsions (Lv et al., 2019). However, prolonged excessive unfolding of protein can reduce the ES (Lv et al., 2019).

#### 3.3.5 Influence on thermal characteristics and thermal stability

TGA is a widely utilized method for investigating thermal characteristics of proteins, with the objective of identifying the ideal processing parameters for food products (Kumar et al., 2014). In our results, two stages of mass loss were observed. The first mass loss (0–100 °C) might be attributed to the previous freeze-drying of the samples (Fig. 4E). The second mass loss (150–400 °C) showed no significant difference between the two extracts, although USBG exhibited a slightly lower weight loss (*P < 0.05*). This result was in agreement with the report of Ortege et al. (2024) on the thermogravimetric curve trends of olive leaf glutelin following UAE. DSC is an effective thermal analysis technique for detecting thermal stability changes of protein (Bai et al., 2023). Our research results indicated that the melting point of USBG (258.7 °C) is significantly higher by 21 °C compared to SBG (237.8 °C) (Fig. 4F). This result was consistent with the slower thermal decomposition trend observed in USBG during TGA. The enthalpy of absorption for USBG (−916 J/g) is only 43 % of that of SBG (−2124 J/g) (*P < 0.05*). The lower absolute value of the enthalpy change, along with the higher melting point, suggested that USBG can more effectively maintain functional integrity under high-temperature conditions (Wang et al., 2020). This synergistic advantage in thermal properties (high melting point + low enthalpy change) makes USBG an ideal choice as a heat-treated food additive, as it can better resist heat-induced denaturation during processing and maintain its functional characteristics.

### 3.4 Effects of UAE on the in vitro digestibility, post-digestion particle size, and amino acid composition of SSM glutelin

The results of the in vitro protein digestibility analysis showed that, compared to SBG, the particle size of USBG decreased by 41.74 % after gastric digestion (Fig. 5B), while its digestibility increased by 22.29 % (Fig. 5A) (*P < 0.05*). After gastrointestinal digestion, the particle size was reduced by 58.57 %, and the digestibility increased by 17.43 %. The reduction of USBG's particle size caused by UAE (Fig. 3E) enhanced the surface area available for enzyme action. Additionally, the alterations in the secondary and tertiary structures of USBG, along with the disruption of disulfide bonds (Fig. 3B, Fig. 3G, Fig. 3H), facilitate easier access to digestive enzymes, thereby enhancing digestibility (Kang et al., 2022). The amino acid analysis indicated that, compared to SBG, USBG showed a 15.41 % increase in total essential amino acid content after gastric digestion, while the total content of non-essential amino acids increased fourfold (Table 2). After gastrointestinal digestion, the total amount of non-essential amino acids in USBG was 2.5 times higher than in SBG (Table 2) (*P < 0.05*). The results were consistent with the trend observed in the effect of UAE on the digestibility of chickpea protein (Kang et al., 2022). UAE induced structural changes to expose active sites within protein molecules, making them more susceptible to digestive enzymes. This increased accessibility contributes to a higher release of amino acids during digestion (Qian et al., 2023). Furthermore, the amino acid content and digestibility after gastrointestinal digestion were both significantly higher than those after gastric digestion. This difference arises because trypsin could more easily break peptide bonds compared to pepsin (Dai et al., 2017).

**Table 2:** Amino acid composition of SSM glutelin after digestion.

Amino acid	SBGGD(mg/100 mg)	USBGGD (mg/100 mg)	SBUG-ID(mg/100 mg)	USBGG-ID(mg/100 mg)
Thr	0.0892 ± 0.00318^b^	0.1315 ± 0.00232^a^	0.2849 ± 0.01473^a^	0.3553 ± 0.0119^a^
Phe	0.1811 ± 0.01268^a^	0.1577 ± 0.00336^a^	ND	ND
Lys	0.0602 ± 0.00202^b^	0.0795 ± 0.00296^a^	2.79 ± 0.11138^a^	2.7014 ± 0.10817^a^
Ile	0.0048 ± 0.00018^a^	0.0091 ± 0.00366^a^	0.0023 ± 0.00036^a^	0.0183 ± 0.00932^a^
Met	ND	ND	0.02 ± 0.00324^a^	0.0146 ± 0.00143^a^
Val	0.0248 ± 0.00717^a^	0.0305 ± 0.00131^a^	0.0836 ± 0.07736^a^	0.126 ± 0.00735^a^
His	0.0312 ± 0.00505^a^	0.0433 ± 0.00212^a^	0.3302 ± 0.01303^a^	0.3327 ± 0.01822^a^
**EAA**	0.3913 ± 0.014268^b^	0.4516 ± 0.04131^a^	3.511 ± 0.00969^a^	3.5483 ± 0.017611^a^
Pro	0.8871 ± 0.76882^b^	7.9941 ± 0.85062^a^	11.2456 ± 1.80649^b^	31.3111 ± 2.67079^a^
Tyr	0.0858 ± 0.2589^a^	0.0901 ± 0.00827^a^	ND	ND
Cys	0.0083 ± 0.00289^a^	0.1186 ± 0.00412^a^	0.2014 ± 0.00788^a^	0.2541 ± 0.00999^a^
Ala	0.0515 ± 0.00252^a^	0.0529 ± 0.00563^a^	0.1498 ± 0.00878^a^	0.1749 ± 0.00656^a^
Gly	0.017 ± 0.02463^a^	0.0025 ± 0.00084^a^	0.0536 ± 0.00159^a^	0.0187 ± 0.00228^a^
Ser	0.0203 ± 0.00208^a^	0.0261 ± 0.00075^a^	0.1558 ± 0.00559^a^	0.164 ± 0.00596^a^
Glu	0.084 ± 0.00509^a^	0.1313 ± 0.00019^a^	0.2561 ± 0.01061^a^	0.373 ± 0.18422^a^
Arg	1.2103 ± 0.20821^a^	1.2932 ± 0.11876^a^	1.2575 ± 0.0917^a^	1.3099 ± 0.11501^a^
**NEAA**	2.439 ± 0.63459^b^	9.7313 ± 0.04723^a^	13.3198 ± 0.00045^b^	33.6057 ± 0.00963^a^

Note: SBG-GD represents gastric digestion of SBG, USBG-GD represents gastric digestion of USBG, SBG-G-ID represents gastro-intestinal two-step digestion of SBG, USBG-G-ID represents gastro-intestinal two-step digestion of USBG, EAA represents total essential amino acids, NEAA represents non-total essential amino acids. ND indicates not detected, and a, b indicate significant differences (*P < 0.05*).

### 3.5 Effects of UAE on the in vitro hypoglycemic and lipid-lowering activity of SSM glutelin

#### 3.5.1 Influence on the inhibitory activities of SSM glutelin against α-amylase and α-glucosidase

**Fig. 6:**
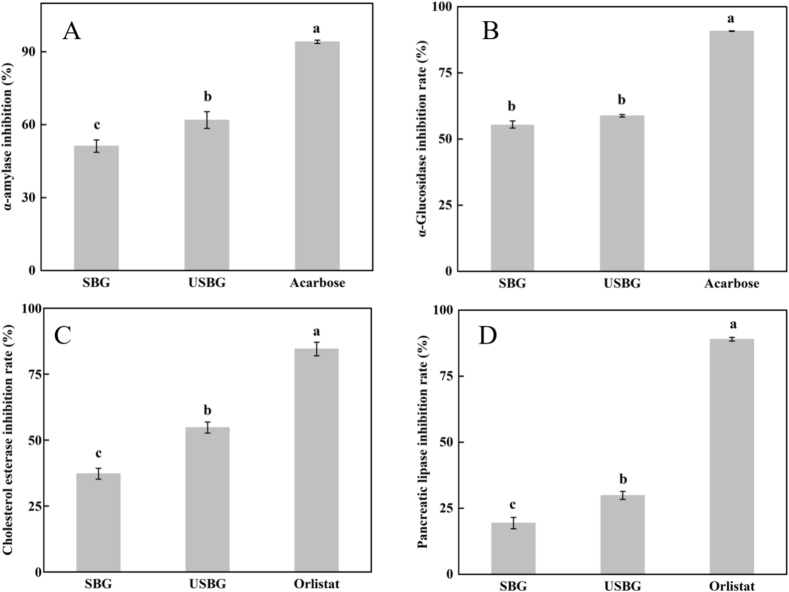
Effects of ultrasound-assisted extraction (UAE) on inhibitory activities of SSM glutelin in terms of α-amylase (A), α-glucosidase (B), pancreatic cholesterol esterase (C), and pancreatic lipase (D). Different letters indicate significant differences between means (*p < 0.05*), while the standard deviation (±SD) was represented by a vertical line.

The pancreas and salivary glands release α-amylase, an essential enzyme (Prakulanon et al., 2015). Inhibiting α-amylase slows the digestion of starch and oligosaccharides, lowering glucose absorption (Cherbal et al., 2017). α-glucosidase is a carbohydrate hydrolase that degrades α-glycosidic bonds. It converts disaccharides, trisaccharides, and oligosaccharides into digestible glucose and other monosaccharides. Inhibiting the activity of this enzyme significantly delays carbohydrate digestion and glucose absorption, reduced the risk of obesity and diabetes (Ghani, 2015). Therefore, these two enzymes were usually used as important indicators for the detection of hypoglycemic activity of compounds in vitro. The results showed that both USBG and SBG had significant inhibitory effects on α-amylase and α-glucosidase. Compared to SBG, the inhibition rate of USBG on α-amylase increased by 20.89 % (Fig. 6A). The inhibition rate of α-glucosidase by USBG was slightly higher than that by SBG (Fig. 6B) (*P < 0.05*). According to the research by Fadimu et al. (2022) after UAE treatment of lupin protein, its hydrolysates exhibited significantly enhanced inhibitory activity against α-glucosidase and α-amylase. The cavitation effect of UAE leads to the dissociation of the protein structure, thereby promoting the release of specific anti-diabetic peptides and enhancing their bioactivity. This research provides new insights for the development of natural antidiabetic functional foods. Therefore, we speculated that both USBG and SBG had hypoglycemic activity, and UAE could improve the hypoglycemic effect of USBG.

#### 3.5.2 Influence on the inhibitory activities of SSM glutelin against pancreatic cholesterol esterase and pancreatic lipase

Cholesterol esterase is a type of polymerase produced by pancreatic acinar cells. It can hydrolyze cholesterol and free fatty acids produced by cholesterol lipids in the food, lowering cholesterol absorption and helping to lower blood lipid levels (Mudgil et al., 2019). Pancreatic lipase is a vital enzyme involved in the absorption and digestion of dietary fat. It can limit fat absorption, lowering the risk of obesity and hyperlipidemia (Birari et al., 2007). Therefore, these two enzymes are often used as important indicators for in vitro detection of lipid-lowering activity of compounds. The results of our study showed that compared with SBG, the inhibition rate of USBG on cholesterol esterase (Fig. 6C) and Pancreatic lipase (Fig. 6D) increased by 47.03 % and 54.03 %, respectively (*P < 0.05*). UAE can disrupt the aggregated state of proteins (such as breaking disulfide bonds or non-covalent interactions) through the cavitation effect, releasing more small peptide fragments, thereby enhancing their competitive binding to the active site of pancreatic lipase (Zhang et al., 2017). Research by Huo et al. (2024) indicated that UAE treatment not only generated more small peptides from walnut meal proteins but also produced more peptides with terminal hydrophobic amino acids, which enhanced their antioxidant activity. Antioxidant activity and lipid-lowering effects are often linked through common pathways. Therefore, we hypothesized that UAE further enhanced lipid-lowering capacity by facilitating the release of hydrophobic bioactive peptides in USBG.

## 4 Conclusion

This study demonstrated that UAE significantly improved the extraction yield and protein content of SSM glutelin. Furthermore, UAE modified the physicochemical characteristics of SSM glutelin by increasing amino acid content, inducing a dispersed and looser structure, decreasing particle size, and altering secondary and tertiary structures. The functional characteristics of SSM glutelin were also enhanced by UAE, as evidenced by increased solubility, water holding capacity, oil holding capacity, foam capacity, oil-in-water emulsion capacity, melting point, and thermal stability. The in vitro digestion results demonstrated that USBG possessed higher digestibility, and higher amino acid content after digestion, which could be attributed to the smaller particle size of USBG compared to SBG during digestion. In vitro activity analysis illustrated that SSM glutelin effectively inhibited α-amylase, α-glucosidase, pancreatic cholesterol esterase and pancreatic lipase activities. Notably, USBG exhibited even stronger hypoglycemic and lipid-lowering activities. According to these results, UAE holds promise for improving SSM glutelin's extraction, structure, functional characteristics, and bioactivities.

## CRediT authorship contribution statement

**Wenxia Dong:** Writing – original draft, Data curation, Conceptualization. **Jinmei Zhao:** Methodology. **Tianyu Zhang:** Software. **Shilong Xiao:** Formal analysis. **Xinyu Wang:** Validation. **Yang Bi:** Writing – review & editing. **Juan Wei:** Supervision, Project administration, Funding acquisition.

## Declaration of competing interest

The authors declare that they have no known competing financial interests or personal relationships that could have appeared to influence the work reported in this paper.

## Data Availability

Data will be made available on request

## Appendix A Supplementary data

Supplementary material. Image 1

## References

[bb0005] Ajayi F.F., Mudgil P., Gan C.-Y., Maqsood S. (2021). Identification and characterization of cholesterol esterase and lipase inhibitory peptides from amaranth protein hydrolysates. Food Chemistry: X.

[bb0010] Alavi F., Chen L., Emam-Djomeh Z. (2021). Effect of ultrasound-assisted alkaline treatment on functional property modifications of faba bean protein. Food Chemistry.

[bb0015] Arzeni, C., Martínez, K., Zema, P., Arias, A., Pérez, O. E., Pilosof, A. M. R. C. (2012). Comparative study of high intensity ultrasound effects on food proteins functionality. *Journal of Food Engineering, 108*, 463-472. doi:10.1016/j.jfoodeng.2011.08.018.

[bb0020] Bai Y., Li X., Xie Y., Wang Y., Dong X., Qi H. (2023). Ultrasound treatment enhanced the functional properties of phycocyanin with phlorotannin from Ascophyllum nodosum. Frontiers in Nutrition.

[bb0025] Birari R.B., Bhutani K.K. (2007). Pancreatic lipase inhibitors from natural sources: Unexplored potential. Drug Discovery Today.

[bb0030] Biswas B., Sit N. (2020). Effect of ultrasonication on functional properties of tamarind seed protein isolates. Journal of Food Science and Technology.

[bb0035] Cherbal A., Kebieche M., Yilmaz E.M., Aydogmus Z., Benzaouia L., Benguessoum M., Madani K. (2017). Antidiabetic and hypolipidemic activities of Algerian Pistachia lentiscus L. leaves extract in alloxan-induced diabetic rats. South African Journal of Botany.

[bb0040] Choi S.-M., Ma C.-Y. (2005). Conformational study of globulin from common buckwheat (*Fagopyrum esculentum Moench*) by Fourier transform infrared spectroscopy and differential scanning calorimetry. J. Agri. Food Chemistry.

[bb0045] Cui Y., Chen J., Zhang S. (2023). The effect of degree of esterification of pectin on the interaction between pectin and wheat gluten protein. Food Hydrocolloids.

[bb0050] Dabbour M., Jiang H., mintah, B. K., Wahia, H., He, R. (2021). Ultrasonic-assisted protein extraction from sunflower meal: Kinetic modeling, functional, and structural traits. Innovative Food Science and Emerging Technologies.

[bb0055] Dai C., Zhang W., He R., Xiong F., Ma H. (2017). Protein breakdown and release of antioxidant peptides during simulated gastrointestinal digestion and the absorption by everted intestinal sac of rapeseed proteins. LWT.

[bb0060] Du H., Zhang J., Wang S., Manyande A., Wang J. (2022). Wang, effect of high-intensity ultrasonic treatment on the physicochemical, structural, rheological, behavioral, and foaming properties of pumpkin (*Cucurbita moschata Duch.*)-seed protein isolates. LWT.

[bb0065] Du J., Zhu Q., Guo J., Wu Y., Hu Z., Yang S., Jiang J. (2023). Effects of ultrasonic and steam-cooking treatments on the physicochemical properties of bamboo shoots protein and the stability of O/W emulsion. Heliyon.

[bb0070] Fadimu G.J., Farahnaky A., Gill H., Truong T. (2022). Influence of ultrasonic pretreatment on structural properties and biological activities of lupin protein hydrolysate. International Journal of Food Science and Technology.

[bb0075] Ghani U. (2015). Re-exploring promising α-glucosidase inhibitors for potential development into oral anti-diabetic drugs: Finding needle in the haystack. European Journal of Medicinal Chemistry.

[bb0080] Ghribi A.M., Gafsi I.M., Blecker C., Danthine S., Attia H., Besbes S. (2015). Effect of drying methods on physicochemical and functional properties of chickpea protein concentrates. Journal of Food Engineering.

[bb0085] Hu H., Fan X., Zhou Z., Xu X., Fan G., Wang L., Huang X., Pan S., Zhu L. (2013). Acid-induced gelation behavior of soybean protein isolate with high intensity ultrasonic pre-treatments. Ultrasonics Sonochemistry.

[bb0090] Hu H., Li-Chan E.C.Y., Wan L., Tian M., Pan S. (2013). The effect of high intensity ultrasonic pre-treatment on the properties of soybean protein isolate gel induced by calcium sulfate. Food Hydrocolloids.

[bb0095] Hu H., Wu J., Li-Chan E.C.Y., Zhu L., Zhang F., Xu X., Pan S. (2013). Effects of ultrasound on structural and physical properties of soy protein isolate (SPI) dispersions. Food Hydrocolloids.

[bb0100] Huang D., Li W., Li G., Zhang W., Chen H., Jiang Y., Li D. (2023). Effect of high-intensity ultrasound on the physicochemical properties of Tenebrio Molitor protein. Food Hydrocolloids.

[bb0105] Huang L., Ding X., Dai C., Ma H. (2017). Changes in the structure and dissociation of soybean protein isolate induced by ultrasound-assisted acid pretreatment. Food Chemistry.

[bb0110] Huo J., Cui Z., Zhang R., Ouyang H., Liu X., Wang P., Yu X., Xie T., Gao S., Li S. (2024). Study on the effect and mechanism of ultrasonic-assisted enzymolysis on antioxidant peptide activity in walnuts. Ultrasonics Sonochemistry.

[bb0115] Indriani S., Benjakul S., Quan T.H., Sitanggang A.B., Chaijan M., Kaewthong P., Karnjanapratum S. (2023). Effect of different ultrasound-assisted process modes on extraction yield and molecular characteristics of pepsin-soluble collagen from Asian bullfrog skin. Food and Bioprocess Technology.

[bb0120] Indriani S., Sae-Leaw T., Benjakul S., Hong Q.T., Karnjanapratum S., Nalinanon S. (2022). Impact of different ultrasound-assisted processes for preparation of collagen hydrolysates from Asian bullfrog skin on characteristics and antioxidative properties. Ultrasonics Sonochemistry.

[bb0125] Jambrak A.R., Lelas V., Mason T.J., Krešić G., Badanjak M. (2009). Physical properties of ultrasound treated soy proteins. Journal of Food Engineering.

[bb0130] Jiang L., Wang J., Li Y., Wang Z., Liang J., Wang R., Chen Y., Ma W., Qi B., Zhang M. (2014). Effects of ultrasound on the structure and physical properties of black bean protein isolates. Food Research International.

[bb0135] Jin J., Okagu O., Yagoub A.E.A., Udenigwe C.C. (2020). Effects of sonication on the in vitro digestibility and structural properties of buckwheat protein isolates. Ultrasonics Sonochemistry.

[bb0140] Julián V., Calderón-Chiu C., Calderón-Santoyo M., Barros-Castillo J.C., López-García M., Ragazzo-Sánchez J.A. (2022). Ultrasound-assisted extraction of Artocarpus heterophyllus L. leaf protein concentrate: Solubility, foaming, emulsifying, and antioxidant properties of protein hydrolysates. Colloids and Interfaces.

[bb0145] Kang S., Zhang J., Guo X., Lei Y., Yang M. (2022). Effects of ultrasonic treatment on the structure, functional properties of chickpea protein isolate and its digestibility in vitro. Foods.

[bb0150] Karaca A.C., Low N., Nickerson M. (2011). Emulsifying properties of canola and flaxseed protein isolates produced by isoelectric precipitation and salt extraction. Food Research International.

[bb0155] Khanizadeh S., Buszard D., Zarkadas C.G. (2019). Misuse of the Kjeldahl method for estimating protein content in plant tissue. HortScience.

[bb0160] Kumar K.S., Ganesan K., Selvraj K., Rao P.V.S. (2014). Studies on the functional properties of protein concentrate of Kappaphycus alvarezii (Doty) Doty – An edible seaweed. Food Chemistry.

[bb0165] Li K., Ma H., Li S., Zhang C., Dai C. (2017). Effect of ultrasound on alkali extraction Protein from Rice dreg flour. Journal of Food Process Engineering.

[bb0170] Li T., Wang L., Geng H., Zhang X., Chen Z. (2021). Formation, structural characteristics, foaming and emulsifying properties of rice glutelin fibrils. Food Chemistry.

[bb0175] Li X., Chen H., Jia Y., Peng J., Li C. (2022). Inhibitory effects against alpha-amylase of an enriched polyphenol extract from pericarp of Mangosteen (*Garcinia mangostana*). Foods.

[bb0180] Li X., Qi B., Zhang S., Li Y. (2023). Effects of ultrasonic treatment on the structural and functional properties of cactus (*Opuntia ficus-indica*) seed protei. Ultrasonics Sonochemisty.

[bb0185] Lin J., Xiang H., Sun-Waterhouse D., Cui C., Wang W. (2022). Deep eutectic solvents and alkaline extraction of protein from seabuckthorn seed meal: A comparison study. Food Science and Human Wellness.

[bb0190] Liu J., Yu X., Liu Y. (2021). Effect of ultrasound on mill starch and protein in ultrasound-assisted laboratory-scale corn wet-milling. Journal of Cereal Science.

[bb0195] Liu Q., Liu Y., Huang H., Xiong M., Yang Y., Lin C., Yang F., Xie Y., Yuan Y. (2023). Improvement of the emulsifying properties of Zanthoxylum seed protein by ultrasonic modification. Ultrasonics Sonochemistry.

[bb0200] Lv S., Taha A., Hu H., Lu Q., Pan S. (2019). Effects of ultrasonic-assisted extraction on the physicochemical properties of different walnut proteins. Molecules.

[bb0205] Moure A., Sineiro J., Dominguez H., Parajo J.C. (2006). Functionality of oilseed protein products: A review. Food Research International.

[bb0210] Mudgil, P., Baby, B., Ngoh, Y-Y., Vijayan, R., Gan, C-Y., Maqsood, S. (2019). Identification and molecular docking study of novel cholesterol esterase inhibitory peptides from camel milk proteins. Journal of Dairy Science*,* 102, 10748–10759. doi:10.3168/jds.2019-16520. 31548068

[bb0215] Oluwajuyitan T.D., Aluko R.E. (2024). Structural and functional properties of fava bean albumin, globulin and glutelin protein fractions. Food Chemistry: X.

[bb0220] Ortega M.L.S., Orellana-Palacios J.C., Garcia S.R., Rabanal-Ruiz Y., Moreno A., Hadidi M. (2024). Olive leaf protein: Extraction optimization, in vitro digestibility, structural and techno-functional properties. International Journal of Biological Macromolecules.

[bb0225] Peng D., He Z., Pan X., Zheng R., Bao H., Liao J., Dong L., Li W., Chen J., Li P., Du B. (2024). A comparative evaluation of the structure, functionality and volatile profiles of Trichosanthes kirilowii seed protein isolates based on different extraction methods. Food Chemistry.

[bb0230] Prakulanon J., Duangsrisai S., Vajrodaya S., Thongchin T. (2015). In vitro evaluation of antioxidant and antidiabetic activities of Syzygium densiflrum fruits. Asian Pacific Journal of Tropical Disease.

[bb0235] Qian J., Chen D., Zhang Y., Gao X., Xu L., Guan G., Wang F. (2023). Ultrasound-assisted enzymatic protein hydrolysis in food processing: Mechanism and parameters. Foods.

[bb0240] Rahel S.D., Brijesh K.T., Farid C., Marco G.-V. (2022). Impact of ultrasound processing in alternative protein systems: Protein extraction, nutritional effects and associated challenges. Ultrasonics Sonochemistry.

[bb0245] Rahman M.M., Lamsal B.P. (2021). Ultrasound-assisted extraction and modification of plant-based proteins: Impact on physicochemical, functional, and nutritional properties. Comprehensive Reviews in Food Science and Food Safety.

[bb0250] Saha J., Deka S.C. (2016). Functional properties of sonicated and non-sonicated extracted leaf protein concentrate from Diplazium esculentum. International Journal of Food Properties.

[bb0255] Sahni P., Sharma S., Surasani V.K.R. (2020). Influence of processing and pH on amino acid profile, morphology, electrophoretic pattern, bioactive potential and functional characteristics of alfalfa protein isolates. Food Chemistry.

[bb0260] Shen X., Fang T., Gao F., Guo M. (2017). Effects of ultrasound treatment on physicochemical and emulsifying properties of whey proteins pre- and post-thermal aggregation. Food Hydrocolloids.

[bb0265] Shi, W., Chen, J., Cui,Y., Zhang, S., Ma, Y., Liu, J. (2024). Heat-induced aggregation behavior of wheat gluten after adding citrus pectin with different esterification degree. Food Hydrocolloids*,* 147. doi:10.1016/j.foodhyd.2023.109420.

[bb0270] Stone A.K., Karalash A., Tyler R.T., Warkentin T.D., Nickerson M.T. (2015). Functional attributes of pea protein isolates prepared using different extraction methods and cultivars. Food Research International.

[bb0275] Vinayashree S., Vasu P. (2021). Biochemical, nutritional and functional properties of protein isolate and fractions from pumpkin (*Cucurbita moschata var. Kashi Harit*) seeds. Food Chemistry.

[bb0280] Wang K., Xu Z., Liao X. (2021). Bioactive compounds, health benefits and functional food products of sea buckthorn: A review. Critical Reviews in Food Science and Nutrition.

[bb0285] Wang Y., Wang Y., Li K., Bai Y., Li B., Xu W. (2020). Effect of high intensity ultrasound on physicochemical, interfacial and gel properties of chickpea protein isolate. LWT.

[bb0290] Xiang H., Waterhouse D.-S., Liu P., Geoffrey W.I.N., Li J., Cui C. (2020). Pancreatic lipase-inhibiting protein hydrolysate and peptides from seabuckthorn seed meal: Preparation optimization and inhibitory mechanism. LWT.

[bb0295] Xiong T., Xiong W., Ge M., Xia J., Li B., Chen Y. (2018). Effect of high intensity ultrasound on structure and foaming properties of pea protein isolate. Food Research International.

[bb0300] Yang J., Huang F., Huang Q., Ma D., Chen Y., Peng D., Yu X., Deng Q., Geng F. (2023). Physical and emulsifying properties of pea protein: Influence of combined physical modification by flaxseed gum and ultrasonic treatment. Food Science and Human Wellness.

[bb0305] Zhang Y., Li J., Li S., Ma H., Zhang H. (2017). Mechanism study of multimode ultrasound pretreatment on the enzymolysis of wheat gluten. Journal of the Science of Food and Agriculture.

[bb0310] Zhao C., Yin H., Yan J., Niu X., Qi B., Liu J. (2021). Structure and acid-induced gelation properties of soy protein isolate–maltodextrin glycation conjugates with ultrasonic pretreatment. Food Hydrocolloids.

[bb0315] Zhao F., Zhang D., Li X., Dong H. (2018). High-pressure homogenization pretreatment before Enzymolysis of soy protein isolate: The effect of pressure level on aggregation and structural conformations of the protein. Molecules.

[bb0320] Zhao Q., Xie T., Hong X., Zhou Y., Fan L., Liu Y., Li J. (2022). Modification of functional properties of perilla protein isolate by high-intensity ultrasonic treatment and the stability of o/w emulsion. Food Chemistry.

[bb0325] Zhou S., Chen W., Fan K. (2024). Recent advances in combined ultrasound and microwave treatment for improving food processing efficiency and quality: A review.*Food*. Bioscience.

[bb0330] Zhu G., Li Y., Xie L., Sun H., Zheng Z., Liu F. (2022). Effects of enzymatic cross-linking combined with ultrasound on the oil adsorption capacity of chickpea protein. Food Chemistry.

[bb0335] Zhu X., Cai L., Liu J., Zhu W., Cui C., Ouyang D., Ye J. (2023). Effect of seabuckthorn seed protein and its arginine-enriched peptides on combating memory impairment in mice. International Journal of Biological Macromolecules.

