# Supplementary material for: Impact of ultrasound-assisted extraction on the functional and structural properties, digestibility, hypoglycemic and lipid-lowering effects of seabuckthorn seed meal glutelin
Source: Food Chem X. 2025 Apr 16;27:102472. doi: 10.1016/j.fochx.2025.102472 (PMC12047606; doi:10.1016/j.fochx.2025.102472)
Supplement: Supplementary file 1 — Supplementary material. [file mmc1.docx]

**Supplementary Material**

**Impact of ultrasound-assisted extraction on the functional and structural properties, digestibility, hypoglycemic and lipid-lowering effects of seabuckthorn seed meal glutelin**

Wenxia Dong^a^, Jinmei Zhao^a^, Tianyu Zhang^a^, Shilong Xiao^a^, Xinyu Wang^a^,

Yang Bi^a^, Juan Wei^a,*^

*^a^ College of Food Science and Engineering, Gansu Agricultural University, Lanzhou 730070, China*

* Correspond author: weijuan@gsau.edu.cn (Juan Wei)

**Fig. S1** Osboren fractionation extraction of protein from seabuckthorn seed meal (SSM). Different letters indicate significant differences between means (*p < 0.05*), while the standard deviation (±SD) was represented by a vertical line.

**Fig. S2** Process flow diagram for the extraction of SSM glutelin

**Table S1** Experimental design and results of process optimization for extracting SSM glutelin using UAE

**Table S2** Significance Testing of the Coefficients in the Response Surface Equation

**Fig. S3** Response surface of four-factor interaction on extraction rate


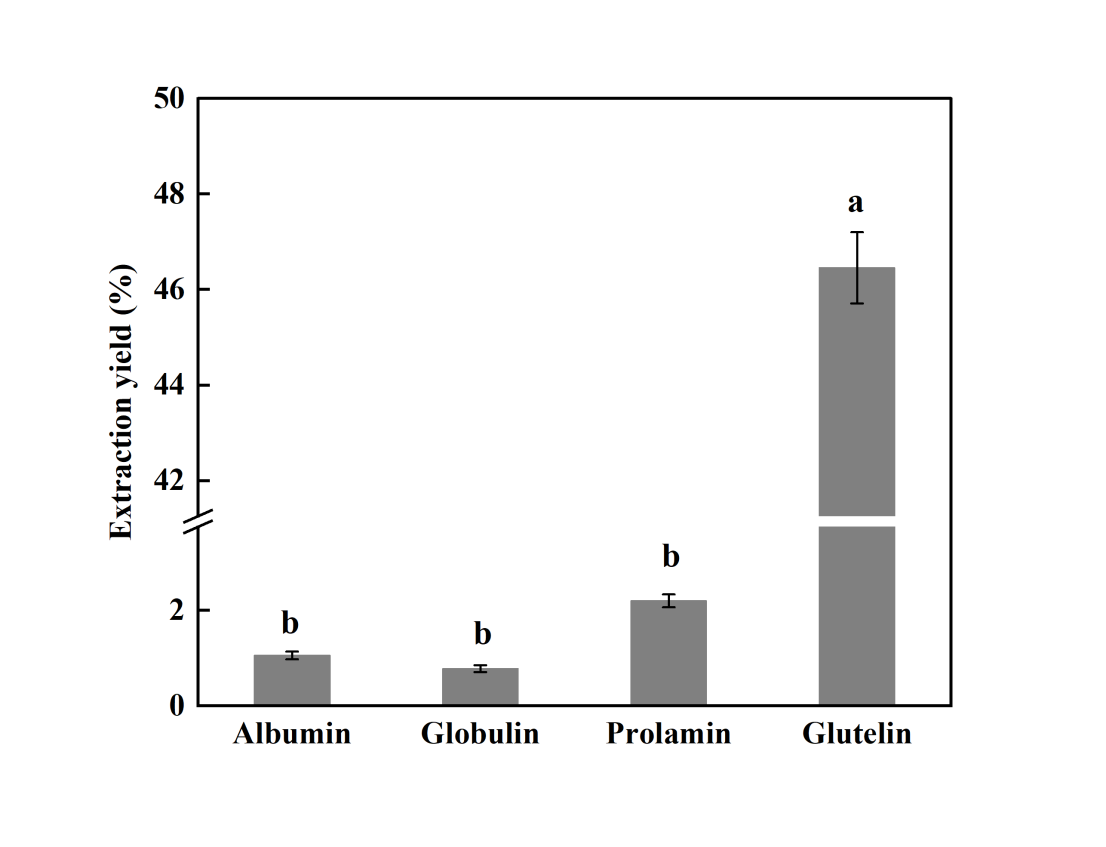


**Fig. S1**

**
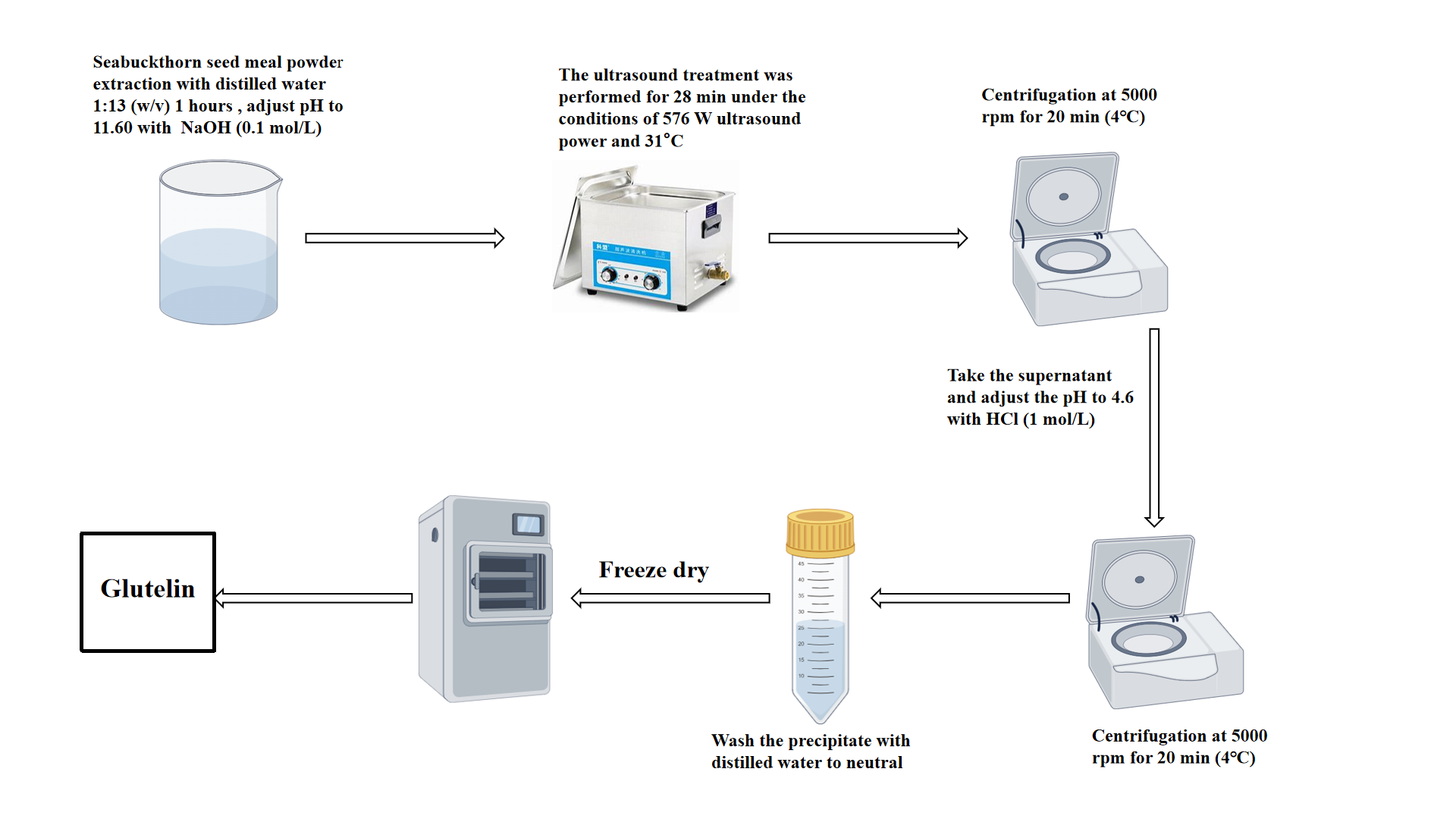
**

**Fig. S2**

**Table S1**

| Run | Factors | | | | Extraction rate（%） |
| --- | --- | --- | --- | --- | --- |
|  | A: pH | B: solid/liquid | C: Time (min) | D: Temp (℃) |  |
| 1 | 10（-1） | 1:14（0） | 10（1） | 30（0） | 26.87 |
| 2 | 11（0） | 1:14（0） | 10（1） | 20（-1） | 17.1 |
| 3 | 10（-1） | 1:12（-1） | 25（0） | 30（0） | 26.2 |
| 4 | 12（1） | 1:12（-1） | 25（0） | 30（0） | 28.21 |
| 5 | 10（-1） | 1:14（0） | 25（0） | 20（-1） | 28.56 |
| 6 | 11（0） | 1:14（0） | 25（0） | 30（0） | 27.56 |
| 7 | 11（0） | 1:14（0） | 10（1） | 40（1） | 18.17 |
| 8 | 10（-1） | 1:14（0） | 25（0） | 40（1） | 24.78 |
| 9 | 11（0） | 1:12（-1） | 25（0） | 40（1） | 27.7 |
| 10 | 10（-1） | 1:16（1） | 25（0） | 30（0） | 25.3 |
| 11 | 11（0） | 1:14（0） | 25（0） | 30（0） | 26.87 |
| 12 | 12（1） | 1:14（0） | 25（0） | 40（1） | 24.38 |
| 13 | 11（0） | 1:12（-1） | 25（0） | 20（-1） | 27.24 |
| 14 | 11（0） | 1:12（-1） | 10（1） | 30（0） | 21.53 |
| 15 | 11（0） | 1:16（1） | 25（0） | 20（-1） | 26.16 |
| 16 | 12（1） | 1:14（0） | 40（-1） | 30（0） | 23.81 |
| 17 | 11（0） | 1:14（0） | 40（-1） | 40（1） | 20.69 |
| 18 | 12（1） | 1:14（0） | 10（1） | 30（0） | 26.87 |
| 19 | 11（0） | 1:16（1） | 10（1） | 30（0） | 26.8 |
| 20 | 11（0） | 1:16（1） | 40（-1） | 30（0） | 24.51 |
| 21 | 11（0） | 1:14（0） | 40（-1） | 20（-1） | 26.87 |
| 22 | 11（0） | 1:14（0） | 25（0） | 30（0） | 27.86 |
| 23 | 12（1） | 1:14（0） | 25（0） | 20（-1） | 18.04 |
| 24 | 11（0） | 1:16（1） | 25（0） | 40（1） | 26.87 |
| 25 | 11（0） | 1:12（-1） | 40（-1） | 30（0） | 19.87 |
| 26 | 11（0） | 1:14（0） | 25（0） | 30（0） | 16.81 |
| 27 | 11（0） | 1:14（0） | 25（0） | 30（0） | 20.42 |
| 28 | 12（1） | 1:16（1） | 25（0） | 30（0） | 26.27 |
| 29 | 10（-1） | 1:14（0） | 40（-1） | 30（0） | 20.58 |

**Table S2**

| Source | Regression coefficient | Degree of freedom | Standard deviation | F-value | P-value | Significance |
| --- | --- | --- | --- | --- | --- | --- |
| Model | 367.95 | 14 | 26.28 | 25.28 | < 0.0001 | *** |
| A-pH | 165.02 | 1 | 165.02 | 158.71 | < 0.0001 | *** |
| B-solid/liquid | 39.89 | 1 | 39.89 | 38.37 | < 0.0001 | *** |
| C-Time | 0.0631 | 1 | 0.0631 | 0.0607 | 0.809 |  |
| D-Temp | 3.75 | 1 | 3.75 | 3.61 | 0.0783 |  |
| AB | 0.2401 | 1 | 0.2401 | 0.2309 | 0.6383 |  |
| AC | 0.1849 | 1 | 0.1849 | 0.1778 | 0.6797 |  |
| AD | 0.4356 | 1 | 0.4356 | 0.4189 | 0.5279 |  |
| BC | 2.1 | 1 | 2.1 | 2.02 | 0.1769 |  |
| BD | 23.33 | 1 | 23.33 | 22.44 | 0.0003 | *** |
| CD | 43.23 | 1 | 43.23 | 41.58 | < 0.0001 | *** |
| A² | 70.19 | 1 | 70.19 | 67.51 | < 0.0001 | *** |
| B² | 4.17 | 1 | 4.17 | 4.01 | 0.0649 |  |
| C² | 0.0947 | 1 | 0.0947 | 0.0911 | 0.7672 |  |
| D² | 29.94 | 1 | 29.94 | 28.79 | < 0.0001 | *** |
| Residual | 14.56 | 14 | 1.04 |  |  |  |
| Lack of Fit | 14.56 | 10 | 1.46 |  |  |  |
| Net error term | 0 | 4 | 0 |  |  |  |
| R^2^ | 0.9619 |  |  |  |  |  |
| Adj-R^2^ | 0.9239 |  |  |  |  |  |
| C·V（%） | 4.21 |  |  |  |  |  |
| Summation | 382.5 | 28 |  |  |  |  |

Note: * * * significant difference (*P* < 0.001); the difference of * * was highly significant (*P* < 0.01); * the difference was significant (*P* < 0.05).


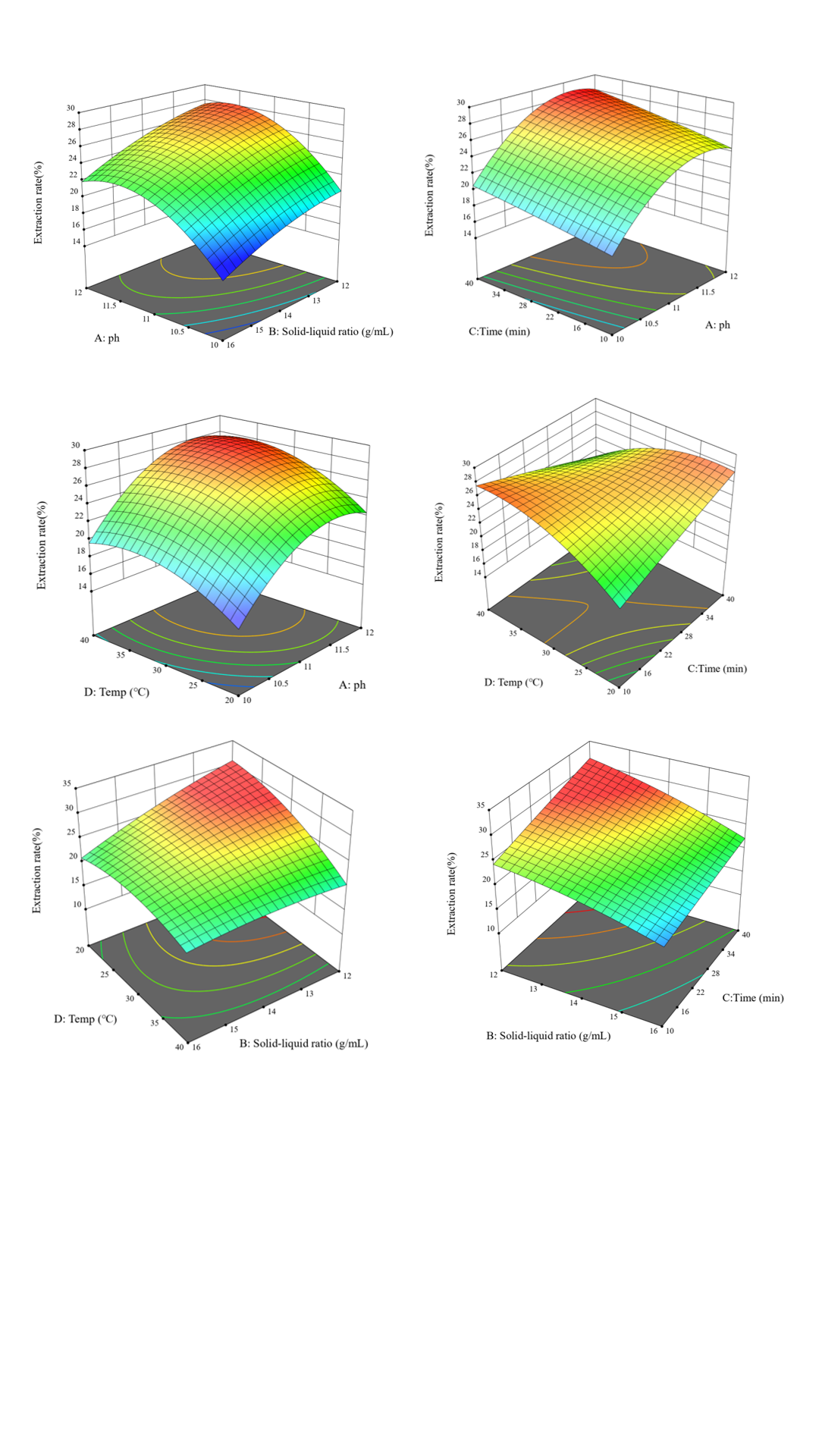


**Fig. S3**
